# Supplementary material for: Commensurate and incommensurate 1D interacting quantum systems
Source: Nat Commun. 2024 Jan 11;15:474. doi: 10.1038/s41467-023-44610-3 (PMC10784295; doi:10.1038/s41467-023-44610-3)
Supplement: Supplementary file 1 — Supplementary Information [file 41467_2023_44610_MOESM1_ESM.pdf]

Supplementary Information

Commensurate and incommensurate 1D interacting quantum systems

Andrea Di Carli, Christopher Parsonage,
Arthur La Rooij, Lennart Koehn, Clemens Ulm,
Callum W Duncan, Andrew J Daley,
Elmar Haller, and Stefan Kuhr

Department of Physics
University of Strathclyde
Glasgow G4 0NG
United Kingdom

1 Supplementary Figures

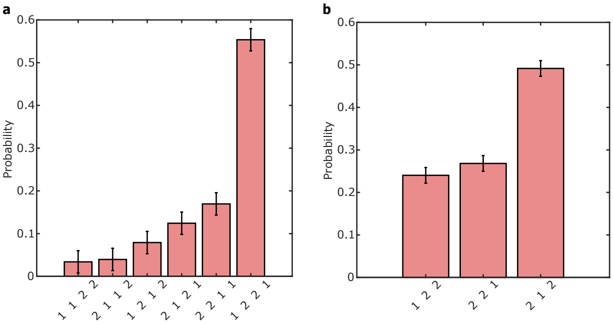

Supplementary Fig. 1 | State detection probabilities for incommensurate systems with two additional particles. a, Probability to detect the different number states for a system with 6 atoms on 4 sites. **b**, same for the system with 5 atoms on 3 sites. Error bars are the standard error.

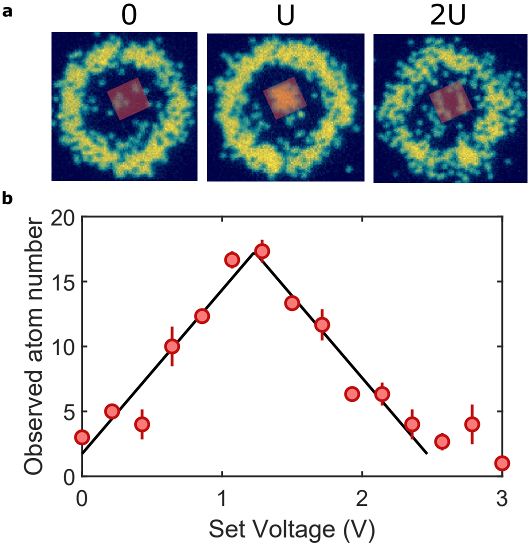

Supplementary Fig. 2 | Calibration of the repulsive barriers. a, Fluorescence image of atoms in a Mott insulating state with an inner $n = 2$ shell (visible as mostly empty sites), illuminated with a square repulsive potential in a region of 5×4 sites, indicated by the red square. For the three images, three different intensities of the 666 nm light were used, corresponding to potential heights of 0, U, and $2U$. The potential depths of the optical lattices were $V_x = V_y = 20(1)\,E_r$ and $V_z = 35(5)\,E_r$, such that $U/h = 940(100)\,\mathrm{Hz}$. **b**, Observed atom number within the region highlighted by the red square in **a**, as function of the set voltage of the laser intensity regulation. Each data point is obtained by averaging the counted atom number in three images, the error bars are standard error. The peak of the graph represents a potential height U at set voltage of 1.23(8)V.

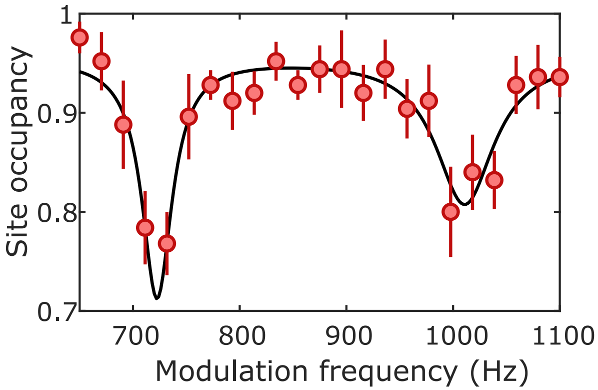

Supplementary Fig. 3 | Calibration of the magnetic gradient field. Observed occupancy within the centre 5×5 sites of a $n = 1$ Mott Insulator at $U/h = 830(100)$ Hz versus the modulation frequency. The line is a fit with a double Lorentzian to obtain the peak positions. Each data point is obtained by averaging over the atom number counted of five images. The error bars are the standard error.

2 Supplementary Methods

2.1 Data analysis and post-selection

We post-select our data sets using two criteria. Firstly, we exclude 1D systems in which we observe an atom on the inner site of the potential wall. For the data presented in Fig. 3, this occurs in less than 5% of systems. When we apply the bias potential (datasets shown in Fig. 5), this rises to around 20% at the maximum potential gradient. Second, we post-select based on the parity of the atom number of each 1D system. When we compress the systems by one site, the parity we observe should be the opposite compared to the initially prepared system since we expect that one site becomes doubly occupied and detected as an empty one.

The fidelity of our system preparation (after Step 1 of the experimental procedure described in Fig. 2a) is such that we observe zero empty sites in typically 75% of cases, an incorrect parity in 20% of cases and two empty sites in 5% of cases. After the whole experimental procedure (Step 4 in Fig. 2a), we find that the observed parity is wrong in 25%-40% of the cases, which we attributed to loss of two atoms and particle-hole pair excitations due to heating from intensity noise of the trapping lasers, especially during the intensity ramps. As the parity is conserved a two-atom loss is not detected in the post-selection. Assuming that the probability to lose an atom in our 1D systems is 0.3, then the probability of losing two atoms is 0.1. We also find that the incommensurate systems require more post-selection indicating that the additional atom is more susceptible to loss which again we attribute to the fact

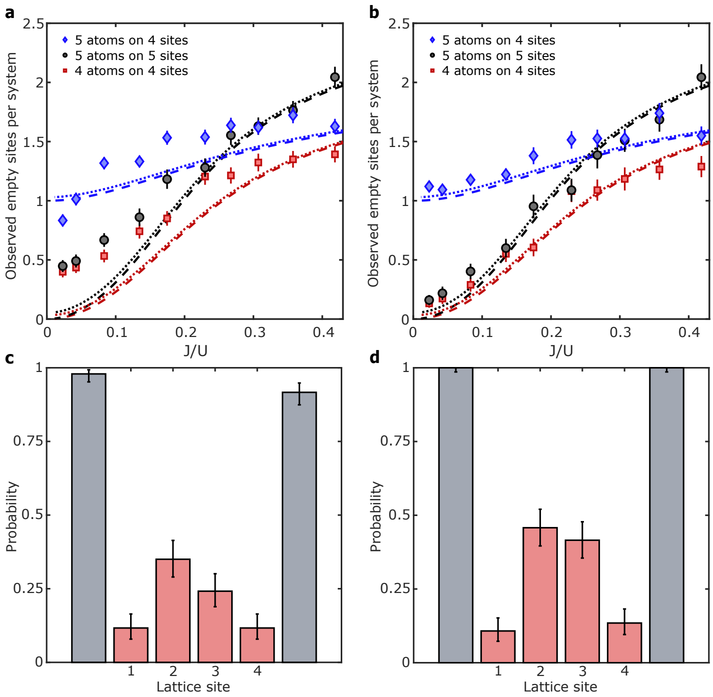

Supplementary Fig. 4 | Effect of post-selection. a, Datasets of Fig. 3a, but without post-selection, **b** same datasets with post-selection for comparison. Error bars in (a) and (b) are the standard error. **c**, probability of detecting empty sites for 5 atoms on 4 sites without post-selection (datasets from Fig. 2i), **d** same datasets after post-selection. Error bars in (c) and (d) are the 95% Clopper-Pearson confidence intervals.

that these systems have a non-gapped excitation spectrum and are more susceptible to technical noise. Overall, we retain approximately 70% of systems for the data sets shown in Fig. 3. For the experiments presented in Fig. 4, when we compress the system by more than one site, we retain on average 60% of the datasets. When applying the bias potential in Fig. 5, we find that about 55% of the incommensurate systems have the correct parity, and 60% – 75% for the commensurate systems.

We illustrate the effect of the post-selection process for selected datasets (Supplementary Fig. 4). For example, in case of the measurement presented in Fig. 3a in the main text, the post-selection process, makes the datasets match the $T = 0$ theory line much better. However a small offset remains due to the fact that we do not post-select systems where the observed atom number is two less than what we would expect. The fraction of the systems with one thermal excitation is typically on the order of 5%.

For all datasets presented in this paper, we have used the eight central 1D systems between the barriers because of their slightly lower entropy.

2.2 Strong-interactions limit

The standard Bose-Hubbard model can be solved in the limit of strong interactions via perturbative approaches, instead of the numerical model we used in this work to simulate the system dynamics. For fixed atom and lattice site numbers, as studied in this work, we can use the limit of $U/J \to \infty$ (deep lattice) to further restrict the finite Hilbert space. From this, analytical solutions for the low-energy states can be found. For example, in the case of 5 atoms on 3 sites, as we take $U/J \to \infty$ it is natural to assume that the possibility of three atoms occupying a single site is vanishingly small due to its high onsite-interaction energy of $6U$. As we have fixed atom and site number, states with two atoms in a single site must be allowed, giving the restricted Hilbert space for the low energy states of $\{|221\rangle, |212\rangle, |122\rangle\}$. The Hamiltonian is then the kinetic energy term only, with the interaction term being a constant diagonal offset for each basis state. The ground state is then given by

$$|\psi\rangle_{\mathrm{GS}}^{5\mathrm{on}3} = \frac{1}{\sqrt{2}}|212\rangle + \frac{1}{2}\left(|221\rangle + |122\rangle\right), \tag{1}$$

with the favouring of the atoms being located on the edge of the system.

A similar process can be repeated for each configuration considered in the main text. For example in the case of 6 atoms on 4 sites, we obtain:

$$\begin{aligned} |\psi\rangle_{\mathrm{GS}}^{6\mathrm{on}4} =& \frac{1}{2}\left(|2121\rangle + |1212\rangle\right) + \frac{1}{\sqrt{5}}\left(|2112\rangle + |1221\rangle\right) \\ &+ \frac{1}{2\sqrt{5}}\left(|2211\rangle + |1122\rangle\right), \end{aligned} \tag{2}$$

and the case of five atoms on four sites

$$|\psi\rangle_{\mathrm{GS}}^{5\mathrm{on}4} = \mathcal{N}\left[|1211\rangle + |1121\rangle + \frac{2}{1+\sqrt{5}}\left(|2111\rangle + |1112\rangle\right)\right], \tag{3}$$

with $\mathcal{N} = (1+\sqrt{5})/\left(2\sqrt{2+\frac{1}{2}\left(1+\sqrt{5}\right)^2}\right)$.

2.3 Details of potential shape

In our numerical simulations we take into account the point spread function of the imaging system, which is calculated assuming it is diffraction limited, causing a broadening of the repulsive potential barriers and an energy offset, ϵ_{off}, on the lattice site closest to the barrier (Supplementary Fig. 5). For a repulsive barrier producing a maximum light shift of $\Delta_{LS}/h = 3.3(5)\,\mathrm{kHz} = 3.5(5)U$, the energy offset is $\epsilon_{\mathrm{off}} = 0.027(4)\Delta_{LS} = 2\pi\hbar \times 90(10)\,\mathrm{Hz}$. This offset becomes significant when we apply the bias potential (measurements presented in Fig. 5) as this energy offset opposes the centre of mass shift. For the lattice depth $V_x = 16(1)\,E_{\mathrm{r}}$ used in Fig. 5, the energy offset on the outermost site of the system is $\epsilon_{\mathrm{off}} \approx 0.1\,U \approx 10J$.

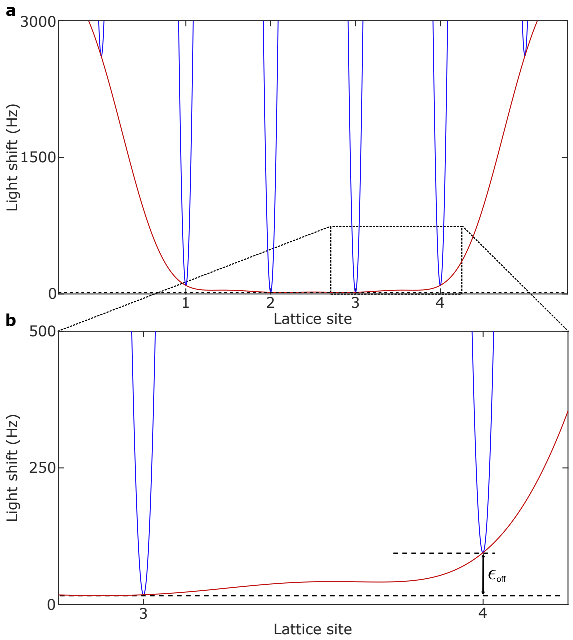

Supplementary Fig. 5 | Effect of the point spread function on the potential shape. a, Lattice potential of the projected repulsive potential barriers (red) and that of the combined potential including the optical lattice (blue). **b**, Magnified view of the outer two lattice sites to show the energy offset ϵ_{off}.
