# Supplementary material for: Commensurate and incommensurate 1D interacting quantum systems
Source: Nat Commun. 2024 Jan 11;15:474. doi: 10.1038/s41467-023-44610-3 (PMC10784295; doi:10.1038/s41467-023-44610-3)
Supplement: Supplementary file 2 — Peer Review File [file 41467_2023_44610_MOESM2_ESM.pdf]

nature portfolio

Peer Review File

Commensurate and incommensurate 1D interacting quantum systems

Open Access This file is licensed under a Creative Commons Attribution 4.0 International License, which permits use, sharing, adaptation, distribution and reproduction in any medium or format, as long as you give appropriate credit to the original author(s) and the source, provide a link to the Creative Commons license, and indicate if changes were made. In the cases where the authors are anonymous, such as is the case for the reports of anonymous peer reviewers, author attribution should be to 'Anonymous Referee' followed by a clear attribution to the source work. The images or other third party material in this file are included in the article's Creative Commons license, unless indicated otherwise in a credit line to the material. If material is not included in the article's Creative Commons license and your intended use is not permitted by statutory regulation or exceeds the permitted use, you will need to obtain permission directly from the copyright holder. To view a copy of this license, visit http://creativecommons.org/licenses/by/4.0/.

Editorial Note: This manuscript has been previously reviewed at another journal that is not operating a transparent peer review scheme. This document only contains reviewer comments and rebuttal letters for versions considered at *Nature Communications*. Mentions of the other journal have been redacted.

REVIEWERS' COMMENTS

Reviewer #1 (Remarks to the Author):

The authors have well taken into account the referee remarks and comments. I was already satisfied with the quality of the presentation, that is now very good. Both the method of compression using the DMD and the results after are interesting. I am thus in favor of publication in nature communication.

Reviewer #2 (Remarks to the Author):

Dear editor,

the authors have adequately addressed my comments that I have raised in my previous report for [redacted]. I am happy to say once more that the paper is of high quality in any respect. The technique is impressive and the results and their description are very clear. I support publication in Nature Communications without hesitation.
